# Global, regional, and national burden of upper respiratory infections, 1990–2021: Findings from the Global Burden of Disease study 2021

**DOI:** 10.1016/j.soh.2024.100084

**Published:** 2024-10-22

**Authors:** Shun-Xian Zhang, Yu-Juan Liu, En-Li Tan, Guo-Bing Yang, Yu Wang, Xiao-Jie Hu, Ming-Zi Li, Lei Duan, Shan Lv, Li-Guang Tian, Mu-Xin Chen, Fan-Na Wei, Qin Liu, Yan Lu, Shi-Zhu Li, Pin Yang, Jin-Xin Zheng

**Affiliations:** a Longhua Hospital, Shanghai University of Traditional Chinese Medicine, Shanghai 200032, China; b NHC Key Laboratory of Parasite and Vector Biology, WHO Collaborating Centre for Tropical Diseases, National Center for International Research on Tropical Diseases, National Institute of Parasitic Diseases, Chinese Center for Disease Control and Prevention (Chinese Center for Tropical Diseases Research), Shanghai 200025, China; c Jinshan Hospital, Fudan University, Shanghai 201508, China; d Gansu Provincial Center for Disease Control and Prevention, Lanzhou 730000, Gansu, China; e Gansu Province People's Hospital, Gansu Provincial Hospital, Lanzhou 730000, Gansu, China; f Georgia Institute of Technology, North Avenue Atlanta, GA 30332, United States; g School of Global Health, Chinese Center for Tropical Diseases Research-Shanghai Jiao Tong University School of Medicine, Shanghai 200025, China

**Keywords:** Global Burden of Disease 2021, Upper respiratory infections, BAPC, One Health

## Abstract

**Background:**

Upper respiratory infections (URIs) are common infectious diseases worldwide. Accurate and timely assessment of the disease burden of URIs is crucial for governments to develop comprehensive prevention and control strategies, and to allocate and utilize healthcare resources more efficiently.

**Methods:**

For URIs in Global Burden of Disease (GBD) 2021 database, age-standardized incidence rates (ASIR), age-standardized prevalence rates (ASPR), age-standardized mortality rates (ASMR), disability-adjusted life-years (DALYs), and case numbers for incidence, prevalence, deaths, and DALYs across the globe, five socio-demographic index (SDI) regions, 21 geographical regions, and 204 countries and territories were provided and analyzed. Trends from 1990 to 2021 were described using the average annual percentage change (AAPC), and future URIs burden was projected with a Bayesian age-period-cohort (BAPC) model.

**Results:**

From 1990 to 2021, there was a significant decline in global ASIR (APCC = −289.86, 95% confidence interval [*CI*]: −298.59 to −281.12), ASPR (AAPC = −4.04, 95% *CI*: −4.16 to −3.92), ASMR (AAPC = −0.02, 95 % *CI*: −0.02 to −0.03) and age-standardized DALY rate (AAPC = −0.75, 95% *CI*: −0.76 to −0.74). The ASIR, ASPR, ASMR, and age-standardized DALY rate were high in elderly for both males and females, and both genders. Similarly, the number of incident cases, prevalence cases, deaths, and DALY cases for URIs was highest in children under five years. The ASMR and age-standardized DALY rate exhibited a negative correlation with SDI across 204 countries and territories in 2021. The ASIR and ASPR for URIs will show an upward trend from 2022 to 2050, while ASMR and age-standardized DALY rate are expected to decline. Low birth weight for gestation remains the leading contributor to deaths related to URIs.

**Conclusion:**

Despite the global decline in URIs burden, significant challenges remain among the elderly population. These findings support the optimization and implementation of public health policies, including targeted vaccination and integrated One Health approaches to reduce the burden in high-risk populations.

## 1 Introduction

Upper respiratory infections (URIs) refer to infectious diseases affecting the upper respiratory tract, including the nasal cavity, pharynx, and larynx [1,2]. The primary pathogens are viruses, such as rhinoviruses, coronaviruses, influenza viruses, and adenoviruses, or bacteria, including *Streptococcus pyogene*s and *Streptococcus pneumoniae*, etc. [2,3]. Clinically, URIs present with symptoms such as nasal congestion, runny nose, cough, sore throat, sneezing, fever, headache, and general fatigue [4,5]. Transmission mainly occurs through respiratory droplets, but infection can also result from contact with contaminated surfaces [6].

URIs are viral in origin, so antibiotic use is generally not recommended. Instead, symptomatic supportive care, including adequate rest, hydration, and antipyretic and analgesic medications, is advised [[7], [8], [9]]. For bacterial infections with severe symptoms, antibiotic therapy may be appropriate under medical supervision [[8], [9], [10]]. Although URIs are common and typically self-limiting, they can lead to serious complications in immunocompromised individuals, such as the elderly, children, and patients with chronic conditions, warranting careful attention [3,11].

Since the 1990s, numerous studies have attempted to estimate the global burden of URIs. However, there remains a lack of consistency in systematic and comparative research on the global burden of URIs [[12], [13], [14]]. The Global Burden of Disease (GBD) 2021 study, which has provided a comprehensive framework for analyzing global health issues since 1990, offers valuable insights. This study uses the newly released GBD 2021 data [[15], [16], [17]], to examine the burden and trends of URIs globally and across individual countries from 1990 to 2021. The findings enhanced our understanding of the epidemiological patterns and public health impact of URIs and offered evidence to inform the optimization of public health policies, resource allocation, and clinical interventions, ultimately aiming to improve population health.

## 2 Methods

### 2.1 Date source

The GBD 2021 is a systematic and comprehensive research initiative aimed at assessing the impact of 371 diseases and injuries, along with 88 risk factors, on population health across the globe, covering 21 regions, 204 countries and territories. The study provides estimates for key burden indicators, including incidence, prevalence, mortality, and disability-adjusted life-years (DALYs), both in terms of absolute numbers and rates. Detailed information on the study's design, data collection, and estimation methods is available in other sources [[15], [16], [17]].

The socio-demographic index (SDI) is a metric used to assess the socio-demographic development level of a country or region, based on three main components: per capita income, educational attainment, and fertility rate. The SDI ranges from 0.00 to 1.00, with countries and regions classified into five development levels: low (< 0.46), low-middle (0.46–0.60), middle (0.61–0.69), high-middle (0.70–0.81), and high (> 0.81) [12,18].

Data were obtained from the Global Health Data Exchange query tool (https://vizhub.healthdata.org), encompassing rates and numbers of URI incidence, prevalence, mortality, and DALYs, along with 95% uncertainty intervals (*UI*s). These data were stratified by sex, region, super-region, and country for the period from 1990 to 2021. According to the World Health Organization's International Classification of Diseases (ICD, 10th Revision), the ICD–10 codes for URIs include J00–J02, J02.8–J03, J03.8–J06.9, J36, and J36.0, while the corresponding ICD–9 codes are 460–465.9, 475–475.9, and 476.9 [17].

### 2.2 Statistical analysis

The percentage changes (PC) in age-standardized incidence rate (ASIR), age-standardized prevalence rate (ASPR), age-standardized mortality rate (ASMR), age-standardized DALY rate, as well as the number of incident cases, prevalence cases, death cases, and DALY cases from 1990 to 2021, were calculated using the following formula [19,20]:

PC = (value_2021_ − value_1990_) / value_2021_ × 100%

Overlapping *UI*s indicated no statistically significant difference (*P* > 0.05), while non-overlapping *UI*s suggested a statistically significant difference (*P* < 0.05).

Smoothing spline models were employed to assess the relationship between the SDI and age-standardized rates (ASRs) and case numbers at global, regional, and national levels, encompassing five SDI regions, 21 geographical regions, and 204 countries and territories [19,20]. Locally weighted scatterplot smoothing was used to determine the optimal degree, number, and location of knots, based on the data characteristics and span parameter. Spearman's rank correlation coefficient was calculated to quantify the strength and direction of the association between dengue rates and SDI [19,20].

The joinpoint regression model provides a comprehensive linear analysis of long-term trends in the ASRs of URIs by segmenting the trends into distinct phases. It calculates the average annual percent change (AAPC) to reflect the overall rate of change across the study period and the annual percent change (APC) to capture variations within specific intervals [13,21]. The formula of the joinpoint regression model is as follows:

$$\text{AP}C_{i}=\{\exp\left(\beta i\right)-1\}\times 100\%$$

$$\text{AAP}C_{i}=\{\exp\left(\frac{\sum W_{i}\beta_{i}}{\sum W_{i}}\right)-1\}\times 100\%$$

The number and location of joinpoints were determined using the Grid Search Method, In the model, *i* is the number of segments, *β*_*i*_ corresponds to the regression coefficients for each linear segment of the data, and *W*_*i*_ represented the length of each corresponding segment. Model selection was optimized through the Monte Carlo permutation test and the modified Bayesian Information Criterion. Both APC and AAPC are unitless measures that indicate the direction and magnitude of trend changes. A negative AAPC or APC, where the upper limit of the 95% confidence intervals (*CI*s) is below zero, signifies a declining trend, while a positive value, with a lower limit above zero, indicates an increasing trend. When neither condition is met, the rate is considered stable, with no significant changes over the period [13].

A Bayesian age-period-cohort (BAPC) model was used to project the cases and ASR of URIs from 2022 to 2050. The BAPC model incorporates the effects of age, period, and birth cohort, where age serves as a primary risk factor for many diseases, while period and cohort capture the influence of other unmeasured factors. The model is structured as a log-linear Poisson model, which assumes multiplicative effects for the age, period, and cohort variables, and is implemented using the R-BAPC and R-INLA packages [22]. The formula of the BAPC model is as follows:

$$\log\left(\lambda_{ij}\right)=\alpha +\mu_{i}+\beta_{j}+\gamma_{k}$$

In this model, *i* (1 ≤ *i* ≤ *I*) represents time points, *j* (1 ≤ *j* ≤ *J*) denotes age groups, *α* represents the intercept, *μ*_*i*_ represents the age effect, *β*_*j*_ represents the period effect, *γ*_*k*_ represents the cohort effect [22].

The estimated annual percentage change (EAPC) was calculated to assess trend fluctuations in ASR of URI from 2022 to 2050. It involved a linear regression model, where the natural logarithm of the rate was regressed on the calendar year, with an independent, normally distributed error term. The EAPC was computed as y=α+βx+ε. Where *y* is equal to natural logarithm of (rate), *x* signifies the calendar year, and *ε* denotes an independent, normally distributed error term. The EAPC and its 95% *CI*s were used to describe trends over specified intervals [*τ*_*j−1*_, *τ*_*j*_]. An EAPC with an upper 95% *CI*s limit below zero indicates a statistically significant declining trend, while a lower 95 % *CI*s limit above zero signifies a significant increasing trend. If the 95% *CI*s include zero, the trend is considered statistically non-significant, suggesting no meaningful change over time [12,23].

All statistical analyses were conducted using R software (version 4.4.1, R Foundation for Statistical Computing, Vienna, Austria; available at https://cran.r-project.org).

## 3 Results

### 3.1 Global epidemiological characteristics

Globally, there has been a significant downward trend in the ASIR (AAPC = −289.86, 95% *CI*: −298.59 to −281.12), ASPR (AAPC = −4.04, 95% *CI*: −4.16 to −3.92), ASMR (AAPC = −0.02, 95% *CI*: −0.02 to −0.03) and age-standardized DALY rate (AAPC = −0.75, 95% *CI*: −0.76 to −0.74) of URIs (Tables 1−4). However, the number of incident cases, prevalence cases, and DALY cases have shown a clear upward trend (Tables S1, S2, S4), while the number of death cases has significantly decreased (Table S3).

**Table 1:** The ASIR of URIs in 1990 and 2021, and changing trends of ASIR across different GBD regions.

Location	ASIR per 100,000 population (95% *UI*) in 1990	ASIR per 100,000 population (95% *UI*) in 2021	Percentage change of ASIR (95% *UI*) in 1990–2021/%	AAPC (95% *CI*) in 1990–2021/%
Global	175,481.29 (156,152.47–198,878.54)	166,770.73 (148,098.16–189,487.93)	−4.96 (−5.44 to −4.41)	−289.86 (−298.59 to −281.12)
East Asia	138,364.40 (121,576.20–158,499.94)	137,200.24 (120,497.82–157,136.25)	−0.84 (−1.59 to −0.13)	−26.15 (−39.63 to −12.67)
Southeast Asia	204,907.06 (181,883.59–231,235.91)	199,235.91 (175,708.01–226,569.61)	−2.77 (−4.77 to −0.31)	−177.95 (−192.22 to −163.68)
Oceania	214,629.64 (189,872.29–245,734.48)	216,592.94 (191,450.24–245,184.51)	0.91 (−2.87 to 4.98)	63.32 (58.46 to 68.18)
Central Asia	104,458.64 (92,328.05–119,543.31)	100,853.61 (89,257.50–115,001.65)	−3.45 (−5.24 to −1.68)	−109.90 (−113.14 to −106.66)
Central Europe	136,076.40 (120,309.51–155,134.10)	135,709.61 (120,162.97–154,473.63)	−0.27 (−1.39 to 0.70)	−12.43 (−13.87 to −10.99)
Eastern Europe	183,839.22 (163,202.87–209,465.29)	183,955.19 (163,129.10–209,785.00)	0.06 (−0.90 to 1.03)	6.53 (0.90 to 12.16)
High-income Asia Pacific	248,805.14 (219,726.02–281,883.78)	247,404.97 (219,448.51–279,609.93)	−0.56 (−1.76 to 0.70)	−52.45 (−68.94 to −35.97)
Australasia	238,899.17 (209,419.01–271,686.46)	237,761.19 (209,660.07–269,978.57)	−0.48 (−4.10 to 3.02)	−39.02 (−47.87 to −30.17)
Western Europe	205,253.85 (180,813.51–232,603.03)	204,141.01 (180,594.83–231,769.20)	−0.54 (−1.59 to 0.45)	−31.60 (−50.98 to −12.23)
Southern Latin America	236,825.22 (209,420.71–267,209.98)	235,918.96 (208,206.18–267,176.12)	−0.38 (−3.07 to 2.69)	−5.85 (−29.09 to 17.38)
High-income North America	332,754.41 (297,062.43–372,909.19)	297,926.78 (265,745.76–333,144.65)	−10.47 (−12.09 to −8.73)	−1262.67 (−1341.98 to −1183.37)
Caribbean	183,721.68 (163,360.20–207,629.13)	186,042.33 (164,416.08–210,791.28)	1.26 (−0.64 to 3.15)	103.92 (84.84 to 123.01)
Andean Latin America	197,571.62 (175,306.43–225,746.13)	196,745.97 (172,869.07–224,104.55)	−0.42 (−2.70 to 2.07)	−36.33 (−50.10 to −22.56)
Central Latin America	195,831.72 (174,236.49–221,306.93)	194,563.33 (171,403.28–221,233.11)	−0.65 (−2.30 to 1.28)	−55.77 (−67.04 to −44.49)
Tropical Latin America	247,639.85 (218,688.87–280,811.11)	242,554.24 (212,927.78–276,375.88)	−2.05 (−3.70 to −0.30)	−161.42 (−171.27 to −151.56)
North Africa and Middle East	165,123.77 (146,650.27–186,469.93)	157,435.27 (139,786.08–179,332.86)	−4.66 (−6.30 to −3.04)	−257.74 (−274.19 to −241.30)
South Asia	143,714.54 (127,187.63–163,440.79)	136,983.62 (121,538.60–156,142.66)	−4.68 (−6.10 to −3.11)	−265.39 (−296.31 to −234.46)
Central Sub-Saharan Africa	172,263.31 (152,175.70–195,647.06)	166,923.37 (147,522.63–188,923.47)	−3.10 (−5.79 to −0.04)	−161.49 (−172.26 to −150.72)
Eastern Sub-Saharan Africa	164,388.69 (145,812.52–186,258.77)	163,889.18 (145,695.78–185,497.29)	−0.30 (−1.55 to 0.91)	−9.01 (−14.12 to −3.90)
Southern Sub-Saharan Africa	227,220.86 (200,202.21–257,155.46)	218,125.61 (194,201.07–246,407.16)	−4.00 (−5.55 to −2.45)	−289.01 (−332.95 to −245.06)
Western Sub-Saharan Africa	143,869.37 (127,639.58–163,520.93)	141,507.19 (125,697.02–161,196.19)	−1.64 (−2.96 to −0.58)	−67.63 (−77.17 to −58.08)
High-middle SDI	165,828.51 (146,841.12–188,311.57)	164,685.58 (146,019.10–187,645.38)	−0.69 (−1.51 to 0.02)	−39.04 (−47.54 to −30.54)
High SDI	244,417.71 (216,965.23–275,014.71)	232,744.64 (206,887.07–261,694.81)	−4.78 (−5.66 to −3.93)	−365.68 (−390.68 to −340.68)
Low-middle SDI	158,350.75 (140,751.46–179,447.94)	153,830.74 (136,960.57–175,052.67)	−2.85 (−3.82 to −1.91)	−169.70 (−185.20 to −154.20)
Low SDI	149,051.76 (132,415.07–169,143.98)	146,602.20 (130,473.03–166,277.37)	−1.64 (−2.58 to −0.71)	−68.87 (−79.56 to −58.19)
Middle SDI	167,801.31 (148,789.98–190,579.31)	165,394.25 (146,461.07–188,308.91)	−1.43 (−2.07 to −0.76)	−82.59 (−87.26 to −77.91)

Abbreviations: AAPC, average annual percent change; ASIR, age-standardized incidence rate; *CI*, confidence interval; GBD, Global Burden of Disease; SDI, socio-demographic index; *UI*, uncertainty interval; URIs, upper respiratory infections.

### 3.2 Epidemiological trends across five SDI regions

From 1990 to 2021, the ASIR, ASPR, ASMR and age-standardized DALY rate for URIs showed a significant downward trend across all five SDI regions. The largest declines in ASIR (AAPC = −365.68, 95% *CI*: −390.68 to −340.68) and ASPR (AAPC = −5.23, 95% *CI*: −5.58 to −4.88) occurred in high SDI regions. In contrast, the greatest reductions in ASMR (AAPC = −0.04, 95% *CI*: −0.04 to −0.03) and the age-standardized DALY rate (AAPC = −1.45, 95% *CI*: −1.47 to −1.43) were observed in middle SDI regions (Tables 1–4).

### 3.3 Epidemiological trends across 21 geographic regions

From 1990 to 2021, the ASIR of URIs increased in Oceania, Eastern Europe, and the Caribbean, with the Caribbean experiencing the largest increase. In Southern Latin America, the ASIR remained relatively stable, while the other 17 regions showed significant declines, with the steepest decline observed in high-income North America (Table 1). The ASPR of URIs increased in Oceania and the Caribbean, remained stable in Eastern Europe, and declined significantly in the remaining 18 regions, with the largest decrease in high-income North America (Table 2). The ASMR showed a significant downward trend across all 21 regions, with the most substantial decline in East Asia (Table 3). Similarly, the age-standardized DALY rate of URIs increased in Oceania, while declining across the other 20 regions, with East Asia experiencing the largest reduction (Table 4).

**Table 2:** The ASPR of URI in 1990 and 2021, and changing trends of ASPR across different GBD regions.

Location	ASPR per 100,000 population (95% *UI*) in 1990	ASPR per 100,000 population (95% *UI*) in 2021	Percentage change of ASPR (95% UI) in 1990–2021/%	AAPC (95% *CI*) in 1990–2021/%
Global	2425.15 (2152.29–2743.08)	2303.67 (2042.29–2612.18)	−5.01 (−5.48 to −4.45)	−4.04 (−4.16 to −3.92)
East Asia	1912.63 (1682.68–2192.49)	1896.35 (1665.56–2168.61)	−0.85 (−1.59 to −0.12)	−0.37 (−0.55 to −0.18)
Southeast Asia	2832.60 (2513.46–3189.45)	2756.67 (2430.72–3134.09)	−2.68 (−4.66 to −0.19)	−2.38 (−2.59 to −2.17)
Oceania	2969.50 (2629.10–3394.84)	2999.12 (2645.74–3386.28)	1.00 (−2.76 to 5.15)	0.86 (0.72 to 1.00)
Central Asia	1438.10 (1272.07–1646.25)	1388.45 (1228.73–1581.78)	−3.45 (−5.25 to −1.64)	−1.51 (−1.56 to −1.47)
Central Europe	1873.52 (1659.24–2135.01)	1868.29 (1653.80–2134.52)	−0.28 (−1.42 to 0.66)	−0.18 (−0.20 to −0.16)
Eastern Europe	2532.22 (2239.45–2875.53)	2533.84 (2241.05–2881.16)	0.06 (−0.90 to 1.06)	0.02 (−0.02 to 0.06)
High-income Asia Pacific	3449.16 (3045.34–3899.70)	3429.84 (3040.90–3872.75)	−0.56 (−1.77 to 0.68)	−0.72 (−0.95 to −0.50)
Australasia	3311.11 (2904.58–3763.63)	3295.38 (2901.28–3736.43)	−0.48 (−4.10 to 3.07)	−0.54 (−0.66 to −0.41)
Western Europe	2845.71 (2510.01–3222.91)	2830.43 (2503.32–3214.22)	−0.54 (−1.53 to 0.43)	−0.43 (−0.70 to −0.17)
Southern Latin America	3283.20 (2910.56–3699.16)	3270.69 (2886.77–3707.98)	−0.38 (−3.05 to 2.56)	−0.34 (−0.60 to −0.07)
High-income North America	4603.86 (4104.77–5145.39)	4128.49 (3687.68–4601.21)	−10.33 (−11.93 to −8.61)	−16.52 (−17.37 to −15.66)
Caribbean	2542.66 (2263.43–2873.89)	2574.86 (2280.80–2917.47)	1.27 (−0.63 to 3.16)	1.46 (1.18 to 1.74)
Andean Latin America	2731.05 (2427.56–3114.52)	2719.71 (2389.51–3082.78)	−0.42 (−2.76 to 1.96)	−0.48 (−0.66 to −0.29)
Central Latin America	2706.19 (2401.25–3056.82)	2689.21 (2370.03–3053.70)	−0.63 (−2.29 to 1.25)	−0.76 (−0.92 to −0.60)
Tropical Latin America	3422.39 (3017.72–3881.82)	3355.70 (2942.34–3822.81)	−1.95 (−3.58 to −0.22)	−2.14 (−2.34 to −1.93)
North Africa and Middle East	2280.44 (2027.75–2574.88)	2173.48 (1931.87–2478.43)	−4.69 (−6.33 to −3.12)	−3.59 (−3.83 to −3.36)
South Asia	1985.73 (1762.54–2258.55)	1888.03 (1675.08–2156.14)	−4.92 (−6.38 to −3.31)	−3.86 (−4.31 to −3.41)
Central Sub-Saharan Africa	2385.10 (2104.44–2703.47)	2310.18 (2043.65–2615.17)	−3.14 (−5.76 to −0.16)	−2.22 (−2.41 to −2.02)
Eastern Sub-Saharan Africa	2271.92 (2018.42–2567.04)	2263.24 (2006.77–2556.80)	−0.38 (−1.59 to 0.80)	−0.17 (−0.25 to −0.09)
Southern Sub-Saharan Africa	3141.34 (2773.81–3563.77)	3019.40 (2681.62–3404.99)	−3.88 (−5.40 to −2.35)	−3.82 (−4.40 to −3.24)
Western Sub-Saharan Africa	1984.04 (1764.01–2254.54)	1951.09 (1732.20–2224.36)	−1.66 (−2.98 to −0.62)	−0.94 (−1.05 to −0.83)
High-middle SDI	2290.78 (2027.24–2602.11)	2275.41 (2018.16–2591.11)	−0.67 (−1.48 to 0.06)	−0.54 (−0.67 to −0.41)
High SDI	3383.60 (3003.89–3807.23)	3224.64 (2867.61–3628.79)	−4.70 (−5.57 to −3.84)	−5.23 (−5.58 to −4.88)
Low-middle SDI	2187.91 (1947.73–2479.71)	2123.38 (1892.47–2414.02)	−2.95 (−3.91 to −2.02)	−2.26 (−2.55 to −1.97)
Low SDI	2059.50 (1835.53–2332.54)	2023.80 (1804.14–2291.68)	−1.73 (−2.68 to −0.82)	−1.00 (−1.15 to −0.84)
Middle SDI	2319.21 (2059.54–2631.33)	2284.75 (2025.49–2597.50)	−1.49 (−2.10 to −0.82)	−1.18 (−1.25 to −1.11)

Abbreviations: AAPC, average annual percent change; ASPR, age-standardized prevalence rate; *CI*, confidence interval; GBD, Global Burden of Disease; SDI, socio-demographic index; *UI*, uncertainty interval; URIs, upper respiratory infections.

**Table 3:** The ASMR of URIs in 1990 and 2021, and changing trends of AMPR across different GBD regions.

Location	ASMR per 100,000 population (95% *UI*) in 1990	ASMR per 100,000 population (95% *UI*) in 2021	Percentage change of ASMR (95% *UI*) in 1990–2021/%	AAPC (95% *CI*) in 1990–2021/%
Global	0.81 (0.23–1.21)	0.28 (0.09–0.61)	−65.50 (−86.47 to −42.19)	−0.02 (−0.02 to −0.03)
East Asia	3.01 (0.57–4.52)	0.15 (0.09–0.36)	−94.92 (−97.70 to −57.81)	−0.09 (−0.09 to −0.09)
Southeast Asia	0.08 (0.02–0.18)	0.03 (0.02–0.05)	−68.69 (−79.66 to −3.72)	−0.01 (−0.02 to −0.00)
Oceania	0.04 (0.00–0.13)	0.02 (0.00–0.08)	−42.07 (−66.52 to −2.17)	−0.01 (−0.02 to −0.00)
Central Asia	0.74 (0.45–1.05)	0.33 (0.23–0.46)	−55.27 (−70.84 to −18.90)	−0.01 (−0.02 to −0.01)
Central Europe	0.15 (0.12–0.17)	0.02 (0.02–0.03)	−84.66 (−87.49 to −79.76)	−0.01 (−0.01 to −0.00)
Eastern Europe	0.47 (0.44–0.51)	0.10 (0.09–0.11)	−79.30 (−81.27 to −77.43)	−0.01 (−0.02 to −0.01)
High-income Asia Pacific	0.49 (0.39–0.60)	0.02 (0.02–0.03)	−96.00 (−96.94 to −93.23)	−0.01 (−0.02 to −0.01)
Australasia	0.07 (0.07–0.08)	0.02 (0.02–0.03)	−68.40 (−72.60 to −63.47)	−0.01 (−0.02 to −0.00)
Western Europe	0.10 (0.10–0.11)	0.02 (0.02–0.03)	−77.37 (−79.21 to −75.14)	−0.01 (−0.02 to −0.00)
Southern Latin America	0.06 (0.06–0.07)	0.01 (0.01–0.02)	−78.56 (−81.78 to −74.25)	−0.01 (−0.02 to −0.00)
High-income North America	0.07 (0.06–0.07)	0.02 (0.02–0.02)	−66.27 (−68.37 to −64.18)	−0.01 (−0.02 to −0.00)
Caribbean	0.16 (0.08–0.30)	0.08 (0.03–0.17)	−48.45 (−69.40 to −8.72)	−0.01 (−0.02 to −0.00)
Andean Latin America	0.36 (0.17–0.68)	0.06 (0.04–0.09)	−83.49 (−89.79 to −69.06)	−0.01 (−0.02 to −0.01)
Central Latin America	0.72 (0.66–0.78)	0.06 (0.05–0.08)	−91.49 (−92.98 to −89.33)	−0.02 (−0.03 to −0.02)
Tropical Latin America	0.15 (0.14–0.16)	0.06 (0.05–0.06)	−62.59 (−67.65 to −56.77)	−0.01 (−0.01 to −0.00)
North Africa and Middle East	0.15 (0.04–0.40)	0.06 (0.04–0.12)	−60.16 (−80.15 to 41.11)	−0.00 (−0.01 to −0.00)
South Asia	0.22 (0.01–0.45)	0.11 (0.02–0.23)	−51.11 (−69.91 to 81.41)	−0.00 (−0.01 to −0.00)
Central Sub-Saharan Africa	1.96 (0.18–4.96)	1.27 (0.09–4.60)	−35.14 (−76.81 to 48.55)	−0.02 (−0.02 to −0.02)
Eastern Sub-Saharan Africa	2.48 (0.18–6.06)	1.58 (0.09–4.17)	−36.38 (−73.62 to 13.57)	−0.03 (−0.04 to −0.03)
Southern Sub-Saharan Africa	0.80 (0.41–1.58)	0.46 (0.28–0.67)	−42.13 (−75.15 to 2.96)	−0.01 (−0.02 to −0.01)
Western Sub-Saharan Africa	1.92 (0.18–5.21)	1.26 (0.14–3.57)	−34.25 (−60.95 to 41.01)	−0.02 (−0.03 to −0.02)
High-middle SDI	0.87 (0.29–1.25)	0.08 (0.06–0.17)	−91.07 (−94.86 to −55.66)	−0.03 (−0.04 to −0.03)
High SDI	0.17 (0.14–0.19)	0.02 (0.02–0.03)	−85.93 (−88.16 to −79.30)	−0.00 (−0.00 to −0.00)
Low-middle SDI	0.41 (0.08–0.78)	0.22 (0.06–0.42)	−45.49 (−60.23 to 10.61)	−0.01 (−0.01 to −0.00)
Low SDI	1.61 (0.11–3.83)	1.10 (0.08–2.78)	−31.68 (−65.55 to 14.47)	−0.02 (−0.02 to −0.02)
Middle SDI	1.35 (0.31–1.99)	0.11 (0.08–0.20)	−91.63 (−95.30 to −58.77)	−0.04 (−0.04 to −0.03)

Notes: The ASMR values are very small, measured in units of one per 100,000, leading to overlap between estimates and their 95 % *CI*s or *UI*s (lower bounds, upper bounds), making the data appear identical. However, if more decimal places were shown, differences between them would become evident. Abbreviations: AAPC, average annual percent change; ASMR, age-standardized mortality rate; *CI*, confidence interval; GBD, Global Burden of Disease; SDI, socio-demographic index; *UI*, uncertainty interval; URIs, upper respiratory infections.

**Table 4:** The age-standardized DALY rate of URIs in 1990 and 2021, and changing trend of age-standardized DALY rate across different GBD regions.

Location	Age-standardized DALY rate per 100,000 population (95 % *UI*) in 1990	Age-standardized DALY rate per 100,000 population (95 % *UI*) in 2021	Percentage change of age-standardized DALY rate (95 % *UI*) in 1990–2021/%	AAPC (95 % *CI*) in 1990–2021/%
Global	100.04 (62.41–140.87)	76.49 (43.52–113.85)	−10.22 (−18.93 to −2.34)	−0.75 (−0.76 to −0.74)
East Asia	138.37 (65.08–187.12)	51.59 (32.03–76.85)	−29.42 (−40.82 to −8.14)	−2.85 (−2.89 to −2.81)
Southeast Asia	74.17 (45.95–110.97)	70.18 (42.76–106.76)	−2.31 (−4.91 to −0.45)	−0.12 (−0.12 to −0.11)
Oceania	74.90 (45.90–113.24)	75.41 (46.11–113.79)	−0.36 (−3.56 to 2.81)	0.03 (0.02 to 0.04)
Central Asia	97.98 (69.57–130.50)	62.24 (44.64–85.17)	4.04 (−19.13 to 34.15)	−1.13 (−1.16 to −1.10)
Central Europe	54.56 (36.33–79.40)	47.56 (29.39–71.63)	−7.83 (−12.67 to −4.74)	−0.22 (−0.23 to −0.22)
Eastern Europe	94.69 (69.70–128.44)	68.81 (43.21–103.26)	−11.53 (−16.51 to −8.24)	−0.84 (−0.85 to −0.82)
High-income Asia Pacific	96.23 (61.67–141.26)	86.85 (52.02–131.68)	−6.32 (−10.96 to −3.45)	−0.30 (−0.31 to −0.29)
Australasia	86.51 (53.47–128.94)	83.22 (50.06–124.15)	−3.06 (−7.34 to 0.71)	−0.10 (−0.11 to −0.10)
Western Europe	77.08 (48.49–114.84)	71.87 (43.55–110.04)	−3.52 (−6.22 to −1.77)	−0.17 (−0.17 to −0.16)
Southern Latin America	85.69 (52.83–128.91)	82.41 (49.89–123.90)	−1.80 (−5.51 to 1.39)	−0.11 (−0.11 to −0.10)
High-income North America	118.81 (72.38–177.38)	103.94 (62.30–154.67)	0.85 (−1.08 to 2.36)	−0.48 (−0.50 to −0.46)
Caribbean	74.07 (47.02–109.78)	70.02 (43.03–104.98)	−4.16 (−11.30 to 0.91)	−0.12 (−0.12 to −0.11)
Andean Latin America	93.01 (61.58–134.83)	71.63 (44.00–108.09)	−9.18 (−21.20 to 0.95)	−0.70 (−0.71 to −0.69)
Central Latin America	113.34 (86.25–150.26)	70.60 (43.58–106.98)	−23.44 (−30.86 to −17.22)	−1.39 (−1.40 to −1.37)
Tropical Latin America	94.32 (59.64–138.25)	86.13 (52.42–130.46)	−5.00 (−8.86 to −2.47)	−0.27 (−0.28 to −0.26)
North Africa and Middle East	62.77 (39.97–92.15)	56.34 (34.90–85.52)	−4.69 (−11.09 to −0.03)	−0.21 (−0.21 to −0.20)
South Asia	61.20 (35.98–89.08)	52.37 (32.54–78.24)	−5.29 (−10.87 to −1.41)	−0.28 (−0.29 to −0.26)
Central Sub-Saharan Africa	166.87 (60.61–334.50)	115.53 (49.90–265.43)	−2.50 (−36.85 to 62.24)	−1.68 (−1.72 to −1.65)
Eastern Sub-Saharan Africa	195.81 (58.76–391.09)	140.21 (48.61–280.47)	−9.25 (−25.76 to 12.24)	−1.77 (−1.81 to −1.72)
Southern Sub-Saharan Africa	113.46 (74.74–169.61)	96.04 (63.52–135.87)	−5.94 (−20.53 to 5.29)	−0.55 (−0.57 to −0.53)
Western Sub-Saharan Africa	158.03 (51.57–362.70)	119.45 (48.46–258.89)	−6.03 (−20.05 to 13.08)	−1.25 (−1.30 to −1.20)
High-middle SDI	94.54 (63.15–130.30)	59.81 (36.79–90.53)	−17.80 (−28.25 to −6.19)	−1.14 (−1.15 to −1.12)
High SDI	90.81 (56.96–135.28)	81.54 (49.19–123.49)	−1.91 (−4.45 to −0.35)	−0.29 (−0.30 to −0.28)
Low-middle SDI	75.70 (44.64–110.28)	65.05 (40.44–94.92)	−4.51 (−11.94 to −1.17)	−0.34 (−0.36 to −0.33)
Low SDI	146.36 (50.54–288.26)	113.63 (44.41–214.34)	−6.15 (−18.98 to −13.97)	−1.04 (−1.08 to −1.01)
Middle SDI	105.76 (68.19–141.75)	61.39 (38.28–92.16)	−21.74 (−31.30 to −8.06)	−1.45 (−1.47 to −1.43)

Abbreviations: AAPC, average annual percent change; *CI*, confidence interval; DALY, disability-adjusted life year; GBD, Global Burden of Disease; SDI, socio-demographic index; *UI*, uncertainty interval; URIs, upper respiratory infections.

### 3.4 Epidemiological trends across 204 countries and territories

From 1990 to 2021, among 204 countries and territories, the ASIR of URIs increased in 31 countries, with the largest rise observed in Rwanda (476.02 per 100,000 population, 95% *UI*: 442.64–509.41). Similarly, the ASPR of URIs increased in 31 countries, with Rwanda again showing the greatest increase (6.14 per 100,000 population, 95% *UI*: 5.71–6.56). The ASMR of URIs rose in 12 countries, with the highest increase in Afghanistan (0.01 per 100,000 population, 95% *UI*: 0.00–0.01). The age-standardized DALY rate of URIs increased in 13 countries, with the Republic of Zimbabwe experiencing the largest rise (0.07 per 100,000 population, 95% *UI*: 0.04–0.11) (Fig. S1, Table S5).

### 3.5 Age-gender epidemiological characteristics

In 2021, there were no significant differences in the ASIR, ASPR, ASMR, or age-standardized DALY rate for URIs between males and females across all 5-year age intervals. However, both the ASIR and ASPR for URIs began to increase steadily from the 20–24 age group in both sexes, while the ASMR and age-standardized DALY rate started to rise gradually in individuals aged 60 years and older. In addition, the highest number of incident cases, prevalence cases, deaths, and DALY cases occurred in children under five years of age (Fig. S2A–D).

### 3.6 Association between ASRs and SDI

In 2021, across 204 countries and territories, the ASIR and ASPR of URIs showed a significant positive correlation with the SDI. In contrast, the ASMR and age-standardized DALY rate exhibited a negative correlation with the SDI (Table 5). The number of cases, including incidence, prevalence, deaths, and DALY cases for URIs, also showed a negative correlation with the SDI.

**Table 5:** The association between the burden of URIs (ASIR, ASPR, ASMR, age-standardized DALY rate) and the SDI.

Location	Index	*r*	*P*
204 countries and territories (2021)	ASIR	0.332	< 0.001
	ASPR	0.333	< 0.001
	ASMR	−0.643	< 0.001
	Age-standardized DALY rate	−0.365	< 0.001
204 countries and territories (2021)	Incident cases	−0.188	0.007
	Prevalence cases	−0.188	0.007
	Death cases	−0.488	< 0.001
	DALYs cases	−0.303	< 0.001
21 geographic regions (global, 1990–2021)	ASIR	0.405	< 0.001
	ASPR	0.403	< 0.001
	ASMR	−0.621	< 0.001
	Age-standardized DALY rate	−0.296	< 0.001
21 geographic regions (global, 1990–2021)	Incident cases	−0.023	0.543
	Prevalence cases	−0.024	0.532
	Death cases	−0.436	< 0.001
	DALYs cases	−0.253	< 0.001

Abbreviations: ASIR, age-standardized incidence rate; ASMR, age-standardized mortality rate; ASPR, age-standardized prevalence rate; DALYs, disability-adjusted life years; SDI, sociodemographic index; URIs, upper respiratory infections.

From 1990 to 2021, the incidence and prevalence of URIs showed no correlation with the SDI in global level. However, the number of deaths and DALY cases of URIs exhibited a significant negative correlation with the SDI (Table 5).

### 3.7 Risk factors for ASMR and age-standardized DALY rate

Among the causes of deaths due to URIs, low birth weight for gestation has consistently been the leading contributor, although its impact has been gradually decreasing. Short gestation for birth weight and household air pollution from solid fuels have remained the second and third leading contributors, respectively, with both also showing a downward trend in recent years (Fig. S3A).

In terms of the DALY numbers attributable to URIs, low birth weight for gestation, short gestation for birth weight, and household air pollution from solid fuels have consistently ranked the first, second, and third, respectively, with their contributions increasing over time (Fig. S3B).

### 3.8 Projecting disease burden

From 2022 to 2050, the ASIR and ASPR for URIs are expected to show an upward trend, while the ASMR and age-standardized DALY rate for URIs are projected to decline (Table 6, Fig. S4A–D).

**Table 6:** Prediction of global disease burden of URIs from 2022 to 2050 years based on the BAPC model.

Index	ASIR	ASPR	ASMR	Age-standardized DALYs rate
Value per 100,000 population (95% *CI*) in 2050	167,853.41 (139,965.73–195,741.08)	2307.31 (1920.76–2693.86)	0.11 (0.02 to 0.20)	51.53 (31.32 to 71.73)
EAPC (95% *CI*) in 2022–2050/%	0.04 (0.03–0.05)	0.03 (0.02–0.04)	−3.15 (−3.17 to −3.12)	−1.34 (−1.37 to −1.32)
AAPC (95% *CI*) in 2022–2050/%	65.16 (63.46–66.85)	0.52 (0.50–0.54)	−0.01 (−0.01 to −0.02)	−0.85 (−0.85 to −0.85)

Abbreviations: AAPC: average annual percent change; ASIR, age-standardized incidence rate; ASMR, age-standardized mortality rate; ASPR, age-standardized prevalence rate; BAPC, Bayesian age-period cohort; *CI*, confidence interval; DALYs, disability-adjusted life years.; EAPC, estimated annual percentage change; URIs, upper respiratory infections.

## 4 Discussion

The results of this study indicate that from 1990 to 2021, the global ASIR, ASPR, ASMR, and age-standardized DALY rate for URIs showed a significant downward trend. However, the number of incident cases, prevalence cases, and DALY cases increased substantially, while the number of death cases declined. The study also found that the ASIR, ASPR, ASMR, and age-standardized DALY rate were higher in older persons compared to younger individuals, adolescents, and children. Notably, these rates increased rapidly among individuals aged 60 years and older.

The ASIR and ASPR of URIs have shown a declining trend globally and in most countries and regions from 1990 to 2021. This decrease can be largely attributed to improvements in sanitation and infrastructure, widespread vaccination, increased public health awareness, and better air quality [12,24]. These factors have collectively reduced the transmission risk and burden of URIs, advancing global disease prevention efforts. However, in some low-income regions, the burden of URIs remains high due to limited resources and public health interventions, with the decline being less pronounced or, in some cases, showing an upward trend.

From 1990 to 2021, the ASMR of URIs declined steadily worldwide and across major regions. This reduction is largely attributed to advances in medical technology and treatment methods, widespread vaccination, improved sanitation, standardized antibiotic and antiviral therapies, increased public health awareness, and strengthened global collaboration in infectious disease response. These factors have collectively reduced the risk of severe cases and complications from URIs, enhancing disease prevention and early intervention efforts, which has significantly lowered URIs-related mortality [25,26].

The study found that the ASIR, ASPR, ASMR, and age-standardized DALY rate for URIs are notably high among individuals aged 60 years and older. This can be attributed to multiple factors. First, the aging immune system, which weakens over time, makes individuals aged 60 and above more vulnerable to infections and related complications. Additionally, older adults often suffer from multiple chronic diseases, such as cardiovascular disease, diabetes, and chronic respiratory conditions, which can worsen the effects of infections like URIs. Age-related physiological changes, including reduced lung function and impaired ability to clear pathogens, further increase the risk. Moreover, older populations may experience delayed diagnosis and limited access to timely healthcare, contributing to higher mortality and disability rates [27,28].

Based on the One Health approach, preventing URIs requires coordinated management of human, animal, and environmental health to reduce transmission risks. This includes promoting vaccination and health education, regulating the use of antibiotics and antiviral drugs, enhancing surveillance of animal diseases and management of livestock environments, and addressing air pollution and improving sanitation facilities. Cross-sector collaboration and data integration are critical for early warning systems and rapid response capabilities. Additionally, developing comprehensive health policies and strengthening international cooperation are essential. These multi-faceted measures can effectively reduce the public health burden of URIs and promote the overall health of humans, animals, and the environment [[29], [30], [31], [32]].

This study has several limitations. It relies on data from the GBD 2021 study, which has substantial gaps in quality and coverage, particularly in developing and low-income countries, where incomplete or inaccurate data may affect the reliability of the findings [[15], [16], [17]]. Additionally, the GBD 2021 estimates are based on modeled rather than direct observational data, potentially leading to overestimation or underestimation of the disease burden [[15], [16], [17]]. Furthermore, the predictive models used, such as the BAPC model, heavily depend on the quality of available data and historical trends. Given that URIs transmission is influenced by factors like climate change and population movement, these models may not fully reflect future variations.

## 5 Conclusion

The study found that both children and the elderly are high-risk groups for URIs. Strengthening vaccination coverage for URIs in children and the elderly, raising public health awareness, and enhancing global infectious disease response efforts are essential measures for further reducing the burden of URIs.

## CRediT authorship contribution statement

**Shun-Xian Zhang:** Writing – review & editing, Writing – original draft, Visualization, Validation, Software, Methodology, Investigation, Formal analysis, Data curation, Conceptualization. **Yu-Juan Liu:** Writing – review & editing, Writing – original draft, Visualization, Validation, Software, Methodology, Investigation, Formal analysis, Data curation, Conceptualization. **En-Li Tan:** Writing – review & editing, Writing – original draft, Visualization, Validation, Software, Methodology, Investigation, Formal analysis, Data curation, Conceptualization. **Guo-Bing Yang:** Validation, Supervision, Software, Methodology, Investigation, Data curation. **Yu Wang:** Visualization, Validation, Software, Methodology, Investigation, Data curation. **Xiao-Jie Hu:** Visualization, Validation, Software, Methodology, Investigation, Data curation. **Ming-Zi Li:** Visualization, Validation, Software, Methodology, Investigation, Data curation. **Lei Duan:** Visualization, Validation, Software, Methodology, Investigation, Data curation. **Shan Lv:** Visualization, Validation, Software, Methodology, Investigation. **Li-Guang Tian:** Validation, Supervision, Software, Methodology, Investigation, Data curation. **Mu-Xin Chen:** Visualization, Validation, Software, Methodology, Investigation, Data curation. **Fan-Na Wei:** Visualization, Validation, Software, Investigation, Data curation. **Qin Liu:** Validation, Supervision, Methodology. **Yan Lu:** Validation, Supervision, Methodology. **Shi-Zhu Li:** Validation, Supervision, Methodology. **Pin Yang:** Writing – review & editing, Writing – original draft, Visualization, Validation, Software, Methodology, Investigation, Formal analysis, Data curation. **Jin-Xin Zheng:** Writing – review & editing, Writing – original draft, Visualization, Validation, Software, Methodology, Investigation, Formal analysis, Data curation.

## Ethics approval and consent to participate

Not applicable.

## Data availability statement

The datasets analyzed during the current study are available at http://ghdx.healthdata.org/gbd-results-tool.

## Consent for publication

All authors consent for publication.

## Funding sources

The study was supported by the fund of 10.13039/100007219Shanghai Natural Science Foundation (grant number 23ZR1464000), and the Talent Fund of Longhua Hospital affiliated to Shanghai University of Traditional Chinese Medicine (grant number LH001.007).

## Declaration of competing interest

The authors declare the following financial interests/personal relationships which may be considered as potential competing interests.
