# Supplementary material for: Global, regional, and national burden of upper respiratory infections, 1990–2021: Findings from the Global Burden of Disease study 2021
Source: Sci One Health. 2024 Oct 22;3:100084. doi: 10.1016/j.soh.2024.100084 (PMC11570330; doi:10.1016/j.soh.2024.100084)
Supplement: Multimedia component 1 [file mmc1.docx]

**Appendix A**

**Global, regional, and national burden of upper respiratory infections, 1990–2021: Findings from the global burden of disease study 2021**

Shun-Xian Zhang ^a, b, 1^, Yu-Juan Liu ^c, 1^, En-Li Tan ^d, 1^, Guo-Bing Yang ^e^, Yu Wang ^a^, Xiao-Jie Hu^a^, Ze-Ming Li ^f^, Lei Duan ^b^, Shan Lv ^b^, Li-Guang Tian ^b^, Mu-Xin Chen ^b^, Fa-Na Wei ^b^, Qin-Liu ^b^, Yan Lu ^b^, Shi-Zhu Li ^b^, Pin Yang ^b *^, Jin-Xin Zheng ^g **^

^a^ Longhua Hospital, Shanghai University of Traditional Chinese Medicine, Shanghai 200032, People's Republic of China.

^b^ NHC Key Laboratory of Parasite and Vector Biology, WHO Collaborating Centre for Tropical Diseases, National Center for International Research On Tropical Diseases, National Institute of Parasitic Diseases, Chinese Center for Disease Control and Prevention (Chinese Center for Tropical Diseases Research), Shanghai 200025, People's Republic of China.

^C^ Jinshan Hospital, Fudan University, Shanghai 201508, People's Republic of China.

^d^ The First Hospital of Lanzhou University, Lanzhou 730000, People's Republic of China.

^e^ Gansu Province People’s Hospital, Gansu Provincial Hospital, Lanzhou 730000, People's Republic of China.

^f^ Georgia institute of technology

^g^ School of Global Health, Chinese Center for Tropical Diseases Research-Shanghai Jiao Tong University School of Medicine, Shanghai 200025, People's Republic of China.

^1^ contributed equally to this work.

* Corresponding author. National Institute of Parasitic Diseases, Chinese Center for Disease Control and Prevention (Chinese Center for Tropical Diseases Research), Shanghai 200025, China.

** Corresponding author. School of Global Health, Chinese Center for Tropical Diseases Research-Shanghai Jiao Tong University School of Medicine, Shanghai 200025, China.

Email addresses: [yangpin_st@163.com](mailto:yangpin_st@163.com) (P. Yang), [jamesjin63@163.com](mailto:jamesjin63@163.com) (J.X. Zheng)

**Items**

Table S1: The incidence cases of URI in 1990 and 2021, and changing trend of incidence cases were analyzed across GBD regions.

Table S2: The prevalence cases of URI in 1990 and 2021, and changing trend of prevalence cases were analyzed across GBD regions.

Table S3: The death cases of URI in 1990 and 2021, and changing trend of death cases were analyzed across GBD regions.

Table S4: The DALY cases of URI in 1990 and 2021, and changing trend of DALY cases were analyzed across GBD regions.

Table S5: The disease burden (ASRI, ASPR, ASMR, age-standardized DALY) of URI across 204 countries and territories, and the changing trend of disease burden were analyzed across 204 countries and territories from 1990 to 2021 year.

Figure S1: The changing trend (AAPC ) of ASR were analyzed across 204 countries and territories.

Figure S2: The ASR of upper respiratory infections showed notable differences across age and gender distributions in 2021 year.

Figure S3: Trends in ASMR and age-standardized DALY rate attributed to upper respiratory infections from 1990 to 2021.

Figure S4: Prediction of global disease burden of upper respiratory infections from 2022–2050 years based on the BAPC model .

**Table S1**

The incidence cases of URIs in 1990 and 2021, and changing trend of incidence cases were analyzed across GBD regions

| Location | Incidence cases (person)  (95% UI).  1990 year | Incidence cases (person)  (95% UI).  2021 year | Percentage change of incidence cases (95% UI). 1990-2021. | AAPC(95%CI). 1990－2021. |
| --- | --- | --- | --- | --- |
| Global | 9683324390.98(8577926599.52,10998717689.68) | 12822251510.84(11429364621.60,14464017730.06) | 32.42(29.56,35.49) | 101060814.63(100529821.72,101591807.54) |
| East Asia | 1697402927.76(1493883021.24,1943520225.71) | 1812276018.41(1600532346.61,2042561816.02) | 6.77(1.88,12.36) | 4262853.24(3794124.56,4731581.92) |
| Southeast Asia | 1020039947.30(895508630.73,1160069130.96) | 1342681233.03(1187125552.15,1521477681.22) | 31.63(27.67,35.90) | 10562828.20(10484542.02,10641114.37) |
| Oceania | 15790666.33(13689528.87,18134774.20) | 32884413.92(28984116.02,37201935.52) | 108.25(99.09,118.68) | 550280.83(548752.27,551809.40) |
| Central Asia | 75193028.18(65935289.28,86365274.76) | 97351160.47(86151447.05,111587141.46) | 29.47(24.99,33.40) | 706129.07(696881.55,715376.58) |
| Central Europe | 165020646.86(146165197.82,186935103.89) | 141761634.37(126086587.17,159140068.76) | -14.09(-17.13,-10.90) | -750645.77(-755857.44,-745434.11) |
| Eastern Europe | 402074031.69(356807218.72,455287608.51) | 348756711.31(312459608.89,391349583.92) | -13.26(-15.53,-10.65) | -1720563.58(-1746171.20,-1694955.97) |
| High-income Asia Pacific | 399890315.18(353671851.54,452714624.16) | 376360748.01(337761881.96,420295994.12) | -5.88(-9.67,-1.83) | -724557.70(-755176.35,-693939.05) |
| Australasia | 46148565.32(40719164.88,52379792.03) | 66039203.58(58633573.62,74221138.85) | 43.10(37.09,49.29) | 640447.68(636920.81,643974.54) |
| Western Europe | 711061710.31(636074536.62,802038386.25) | 760418285.56(682315601.94,848171030.15) | 6.94(4.48,9.46) | 1582855.13(1540120.11,1625590.15) |
| Southern Latin America | 118609064.58(104781934.50,133935245.59) | 148642881.43(131781895.49,168090710.38) | 25.32(20.47,30.64) | 978705.31(970686.42,986724.20) |
| High-income North America | 893418600.77(799544514.70,1002665658.63) | 996705967.41(893555063.64,1099824801.96) | 11.56(8.15,15.21) | 2869182.57(2661809.23,3076555.90) |
| Caribbean | 67663370.85(59790918.58,77065491.45) | 84598433.67(75041384.10,95507132.92) | 25.03(21.12,28.92) | 558962.99(549991.07,567934.92) |
| Andean Latin America | 82334890.99(72137526.68,95377205.24) | 129366351.34(113621189.60,146994728.62) | 57.12(51.00,63.52) | 1506298.14(1485149.48,1527446.81) |
| Central Latin America | 351865635.89(309464187.50,401573680.51) | 477980673.88(423733832.32,544460189.10) | 35.84(31.28,40.83) | 4062803.47(4009381.54,4116225.40) |
| Tropical Latin America | 399578235.86(349281210.05,456041158.12) | 518444969.28(457996279.81,589427096.29) | 29.75(24.70,34.66) | 3869980.09(3844303.64,3895656.55) |
| North Africa and Middle East | 615226551.89(541145031.58,705831958.07) | 991721548.07(878516511.83,1137535116.38) | 61.20(55.40,67.60) | 12280577.19(12207646.75,12353507.62) |
| South Asia | 1711743896.93(1492080164.75,1964029794.11) | 2533926864.14(2237452472.40,2904035994.89) | 48.03(42.28,53.95) | 25851184.27(25051504.36,26650864.18) |
| Central Sub-Saharan Africa | 110334874.71(95775725.41,127306603.70) | 255162795.73(221357275.12,292829779.83) | 131.26(123.97,139.74) | 4722347.27(4693337.09,4751357.45) |
| Eastern Sub-Saharan Africa | 362887037.35(317773404.12,418030714.00) | 769176385.39(673058103.79,881752994.75) | 111.96(107.81,116.25) | 13135890.52(13098936.74,13172844.31) |
| Southern Sub-Saharan Africa | 129631715.77(113056401.04,148261153.32) | 178125998.92(157664649.84,202432075.24) | 37.41(33.06,41.84) | 1596898.04(1567479.04,1626317.04) |
| Western Sub-Saharan Africa | 307408676.45(269358074.49,353912447.66) | 759869232.91(666555153.62,876984481.50) | 147.19(141.78,151.17) | 14679497.56(14592405.51,14766589.61) |
| High-middle SDI | 1739416355.47(1546335914.04,1970157460.27) | 1932825530.58(1731026517.16,2168128766.76) | 11.12(7.67,15.16) | 6217926.99(6136773.48,6299080.50) |
| High SDI | 2022724980.34(1813206921.70,2268831452.61) | 2226805415.86(2006360950.13,2466635973.11) | 10.09(7.36,13.08) | 6464708.92(6349043.38,6580374.45) |
| Low-middle SDI | 2020593630.20(1771480196.71,2309522469.43) | 3007917617.91(2670942468.53,3441303026.17) | 48.86(44.96,53.00) | 31808916.47(31274930.17,32342902.77) |
| Low SDI | 848842605.10(744036493.32,975063019.16) | 1792029761.25(1571542474.89,2059434868.59) | 111.11(107.27,114.88) | 30630579.17(30488570.50,30772587.84) |
| Middle SDI | 3043117148.88(2677196077.68,3473973325.21) | 3852100880.34(3423764453.95,4372444512.76) | 26.58(22.30,31.35) | 25966515.34(25698333.14,26234697.54) |

Notes: AAPC, average annual percent change; CI, Confidence interval; GBD, Global Burden of Disease; SDI, Sociodemographic Index; UI, Uncertainty interval; URI, upper respiratory infections.

**Table S2**

The prevalence cases of URIs in 1990 and 2021, and changing trend of prevalence cases were analyzed across GBD regions

| Location | Prevalence cases (person)  (95% UI).  1990 year | Prevalence cases (person)  (95% UI).  2021 year | Percentage change of prevalence cases (95% UI). 1990-2021. | AAPC(95%CI). 1990－2021. |
| --- | --- | --- | --- | --- |
| Global | 133876831.24(118645148.99,151913677.59) | 177076278.89(157506165.96,199365026.69) | 32.27(29.45,35.34) | 1391002.17(1383290.64,1398713.70) |
| East Asia | 23452195.78(20684135.87,26856142.71) | 25029973.48(22135439.09,28238640.61) | 6.73(1.92,12.23) | 58247.83(51980.04,64515.61) |
| Southeast Asia | 14115242.15(12400739.10,16059339.88) | 18568967.55(16412019.43,21010178.09) | 31.55(27.61,35.77) | 145862.06(144778.99,146945.13) |
| Oceania | 218748.03(189379.22,250400.18) | 455668.79(400813.31,516129.07) | 108.31(99.36,119.12) | 7628.57(7606.96,7650.18) |
| Central Asia | 1035486.92(910956.55,1189324.10) | 1340234.31(1187863.72,1529756.77) | 29.43(24.88,33.44) | 9880.00(9741.41,10018.58) |
| Central Europe | 2271673.92(2016846.92,2571755.56) | 1951031.24(1733317.24,2189976.42) | -14.11(-17.12,-10.89) | -10245.47(-10332.03,-10158.91) |
| Eastern Europe | 5537256.07(4916739.01,6271877.01) | 4802119.20(4305453.53,5382201.05) | -13.28(-15.55,-10.51) | -23724.12(-24086.62,-23361.62) |
| High-income Asia Pacific | 5531695.50(4892698.15,6268530.38) | 5198556.59(4658100.08,5798435.86) | -6.02(-9.80,-1.98) | -10123.92(-10551.27,-9696.58) |
| Australasia | 638615.13(563789.04,725666.11) | 913234.69(811356.65,1026713.68) | 43.00(37.06,49.18) | 8843.42(8794.63,8892.21) |
| Western Europe | 9834259.19(8786187.50,11059717.71) | 10513591.28(9442526.17,11725279.75) | 6.91(4.50,9.48) | 21812.49(21231.00,22393.99) |
| Southern Latin America | 1644756.63(1455204.12,1854538.99) | 2057338.44(1821433.98,2330919.84) | 25.08(20.39,30.45) | 13418.62(13305.30,13531.94) |
| High-income North America | 12344872.33(11067637.45,13855586.65) | 13774249.14(12370837.47,15193821.07) | 11.58(8.24,15.14) | 39904.70(37021.28,42788.12) |
| Caribbean | 936606.79(828870.69,1064922.79) | 1169957.55(1037386.73,1321662.53) | 24.91(21.04,28.84) | 7451.38(7343.37,7559.39) |
| Andean Latin America | 1139215.25(997654.39,1316021.65) | 1788021.45(1567595.46,2028347.92) | 56.95(51.02,62.94) | 20906.19(20604.14,21208.23) |
| Central Latin America | 4866768.18(4278786.74,5568595.77) | 6604933.48(5838026.77,7497627.08) | 35.71(31.20,40.68) | 55989.81(55228.63,56751.00) |
| Tropical Latin America | 5528668.82(4840769.90,6296560.23) | 7164582.80(6333145.01,8133053.43) | 29.59(24.72,34.49) | 53302.80(52990.17,53615.42) |
| North Africa and Middle East | 8508427.22(7476015.11,9755124.71) | 13693657.74(12132426.36,15656988.02) | 60.94(55.33,67.34) | 169052.12(168073.34,170030.90) |
| South Asia | 23681325.55(20730783.77,27134776.50) | 34922392.97(30808442.26,39962249.01) | 47.47(41.67,53.36) | 365669.13(359642.16,371696.09) |
| Central Sub-Saharan Africa | 1530697.51(1332668.24,1769382.29) | 3539308.49(3076007.63,4058248.07) | 131.22(124.29,139.41) | 65545.02(65154.43,65935.61) |
| Eastern Sub-Saharan Africa | 5022879.67(4398866.82,5767605.34) | 10636031.02(9320749.61,12182664.88) | 111.75(107.76,115.99) | 181532.32(180995.58,182069.07) |
| Southern Sub-Saharan Africa | 1794569.59(1566275.82,2044264.14) | 2465991.99(2180602.88,2800569.28) | 37.41(33.12,41.83) | 22155.13(21747.24,22563.01) |
| Western Sub-Saharan Africa | 4242870.98(3721325.22,4871376.17) | 10486436.69(9227701.98,12110624.35) | 147.15(141.72,151.11) | 202581.29(201300.63,203861.94) |
| High-middle SDI | 24018061.09(21351952.21,27142094.78) | 26675716.71(23832081.27,29963977.27) | 11.07(7.68,15.09) | 85440.80(84303.92,86577.69) |
| High SDI | 27958499.77(25017559.13,31414317.52) | 30766276.22(27752756.19,34104595.67) | 10.04(7.37,12.95) | 88888.60(87289.62,90487.58) |
| Low-middle SDI | 27952946.77(24588400.94,31979953.81) | 41525308.71(36821656.50,47404689.47) | 48.55(44.60,52.79) | 438990.02(434130.11,443849.92) |
| Low SDI | 11746038.48(10285849.16,13459517.32) | 24764481.15(21752998.33,28444909.63) | 110.83(106.95,114.59) | 422058.18(419846.32,424270.05) |
| Middle SDI | 42082040.18(37032030.57,48049497.12) | 53198407.04(47266593.12,60339356.88) | 26.42(22.21,31.17) | 356624.16(352392.34,360855.99) |

Notes: AAPC, average annual percent change; CI, Confidence interval; GBD, Global Burden of Disease; SDI, Sociodemographic Index; UI, Uncertainty interval; URI, upper respiratory infections.

**Table S3**

The death cases of URIs in 1990 and 2021, and changing trend of death cases were analyzed across GBD regions

| Location | Death cases (person)  (95% UI).  1990 year | Death cases (person)  (95% UI).  2021 year | Percentage change of death cases (95% UI). 1990-2021. | AAPC(95%CI). 1990－2021. |
| --- | --- | --- | --- | --- |
| Global | 37049.26(10999.82,59643.97) | 19611.49(7037.53,41577.15) | -47.07(-77.15,-16.25) | -567.85(-584.29,-551.41) |
| East Asia | 17931.74(3554.30,26516.63) | 2330.54(1381.61,5520.21) | -87.00(-94.17,3.39) | -521.08(-530.22,-511.95) |
| Southeast Asia | 291.22(87.54,566.33) | 172.17(107.78,331.85) | -40.88(-60.83,92.93) | -3.85(-3.91,-3.79) |
| Oceania | 1.23(0.11,3.89) | 1.73(0.21,6.17) | 40.79(-19.11,130.11) | 0.02(0.01,0.02) |
| Central Asia | 669.70(400.07,959.11) | 323.42(221.71,446.74) | -51.71(-69.00,-10.69) | -11.31(-11.57,-11.04) |
| Central Europe | 156.79(132.54,176.77) | 36.72(30.51,46.78) | -76.58(-80.48,-70.65) | -3.80(-3.88,-3.72) |
| Eastern Europe | 928.56(858.72,996.15) | 209.36(192.84,225.84) | -77.45(-79.42,-75.23) | -22.48(-23.14,-21.82) |
| High-income Asia Pacific | 686.48(564.88,856.97) | 111.93(81.51,171.24) | -83.69(-87.99,-72.36) | -18.87(-19.29,-18.46) |
| Australasia | 13.37(12.33,14.49) | 9.09(7.81,10.45) | -32.06(-41.82,-21.82) | -0.11(-0.14,-0.08) |
| Western Europe | 398.32(367.14,426.13) | 171.40(148.44,187.61) | -56.97(-61.33,-53.27) | -7.27(-7.53,-7.02) |
| Southern Latin America | 29.92(26.63,33.78) | 10.00(8.83,11.53) | -66.57(-71.68,-59.62) | -0.61(-0.65,-0.57) |
| High-income North America | 187.92(175.46,198.70) | 96.30(87.87,103.56) | -48.76(-51.97,-45.58) | -3.03(-3.14,-2.92) |
| Caribbean | 58.27(27.33,114.24) | 35.68(13.56,72.32) | -38.76(-62.97,6.90) | -0.68(-0.71,-0.65) |
| Andean Latin America | 157.64(73.09,306.07) | 35.32(22.73,52.42) | -77.59(-86.51,-56.97) | -3.93(-4.00,-3.87) |
| Central Latin America | 1257.27(1141.47,1384.02) | 136.15(111.80,167.64) | -89.17(-91.06,-86.46) | -37.04(-37.90,-36.19) |
| Tropical Latin America | 204.02(184.79,224.50) | 123.12(110.52,135.96) | -39.65(-47.66,-29.55) | -2.76(-2.94,-2.59) |
| North Africa and Middle East | 370.92(113.16,849.87) | 281.24(169.61,557.79) | -24.18(-60.15,177.09) | -2.78(-2.92,-2.63) |
| South Asia | 2250.33(109.78,4914.70) | 1565.73(295.78,3294.95) | -30.42(-54.41,185.05) | -22.34(-23.56,-21.13) |
| Central Sub-Saharan Africa | 1224.34(120.43,3200.64) | 1328.00(126.55,4555.92) | 8.47(-65.66,176.74) | 3.02(2.62,3.41) |
| Eastern Sub-Saharan Africa | 5501.51(413.22,13357.42) | 6001.40(422.87,15927.73) | 9.09(-60.70,114.93) | 14.11(11.86,16.35) |
| Southern Sub-Saharan Africa | 324.68(168.10,599.57) | 315.35(191.37,462.14) | -2.87(-57.61,69.51) | -0.25(-0.40,-0.10) |
| Western Sub-Saharan Africa | 4405.02(416.59,13269.28) | 6316.84(813.00,18270.64) | 43.40(-18.00,204.21) | 63.16(61.12,65.20) |
| High-middle SDI | 7277.37(2528.03,10246.61) | 1203.14(863.65,2736.83) | -83.47(-90.69,-15.50) | -201.65(-205.24,-198.07) |
| High SDI | 1557.07(1286.93,1816.66) | 411.52(337.48,643.78) | -73.57(-78.51,-56.30) | -37.08(-37.59,-36.56) |
| Low-middle SDI | 4405.70(863.04,9291.34) | 3582.65(1054.98,6839.18) | -18.68(-42.19,66.20) | -25.51(-27.02,-23.99) |
| Low SDI | 9728.54(698.73,24329.06) | 12079.86(1099.23,31489.12) | 24.17(-41.57,126.91) | 76.71(73.79,79.62) |
| Middle SDI | 14070.98(4174.05,20126.04) | 2326.55(1655.76,4166.98) | -83.47(-90.52,-36.03) | -386.46(-393.07,-379.85) |

Notes: AAPC, average annual percent change; CI, Confidence interval; GBD, Global Burden of Disease; SDI, Sociodemographic Index; UI, Uncertainty interval; URI, upper respiratory infections.

**Table S4**

The DALY cases of URIs in 1990 and 2021, and changing trend of DALY cases were analyzed across GBD regions

| Location | DALYs cases (person)  (95% UI).  1990 year | DALYs cases (person)  (95% UI).  2021 year | Percentage change of DALY cases (95% UI). 1990-2021. | AAPC(95%CI). 1990－2021. |
| --- | --- | --- | --- | --- |
| Global | 5603818.48(3445643.37,7908213.66) | 5678886.77(3262480.29,8381211.86) | -2.48(-11.57,6.20) | 927.54(6.68,1848.41) |
| East Asia | 1456508.20(751474.17,1978036.03) | 672330.75(414926.09,1006017.36) | -31.07(-43.17,-8.21) | -26330.28(-26816.41,-25844.16) |
| Southeast Asia | 369260.53(227138.92,555358.91) | 472289.55(287300.31,718396.65) | 10.27(7.45,12.76) | 3387.74(3357.58,3417.89) |
| Oceania | 5514.79(3320.81,8488.50) | 11475.39(6973.41,17435.74) | 25.87(21.42,30.51) | 192.02(191.51,192.53) |
| Central Asia | 83742.14(58010.43,111402.10) | 60803.93(43695.37,83393.63) | -10.85(-32.87,16.57) | -750.10(-774.01,-726.20) |
| Central Europe | 64305.31(42216.75,93326.80) | 49084.61(30159.86,72848.18) | -11.81(-16.41,-8.35) | -508.59(-524.23,-492.94) |
| Eastern Europe | 192604.86(138763.99,265406.89) | 127047.83(80587.97,188519.03) | -19.19(-25.07,-15.02) | -2130.59(-2167.91,-2093.28) |
| High-income Asia Pacific | 152321.08(97643.59,223650.49) | 130719.25(80058.86,196025.81) | -4.96(-8.69,-2.30) | -674.92(-695.86,-653.99) |
| Australasia | 16554.83(10228.58,24656.00) | 22871.50(13976.50,34224.86) | 6.45(1.98,10.74) | 204.02(202.12,205.91) |
| Western Europe | 261336.94(164612.69,387500.25) | 264264.76(162028.23,395419.73) | -1.50(-3.73,0.35) | 88.47(70.84,106.10) |
| Southern Latin America | 42952.69(26424.07,64494.15) | 51592.07(31234.94,77340.32) | 6.70(2.52,10.04) | 281.46(278.19,284.72) |
| High-income North America | 316621.14(193859.92,471079.76) | 343018.92(207730.83,506477.74) | 9.88(7.56,12.23) | 699.46(631.99,766.92) |
| Caribbean | 27632.13(17296.15,41132.97) | 31469.40(19450.66,47496.52) | 4.64(-4.19,10.39) | 123.87(118.71,129.03) |
| Andean Latin America | 41257.19(26959.65,59786.91) | 46932.44(28722.82,70944.75) | 3.48(-11.98,17.83) | 175.18(164.75,185.62) |
| Central Latin America | 221600.41(172285.79,288073.41) | 172092.23(106001.99,261492.07) | -16.02(-25.53,-7.87) | -1614.56(-1640.11,-1589.01) |
| Tropical Latin America | 152744.32(95480.35,225172.32) | 182922.22(111736.82,274684.07) | 7.37(3.45,10.24) | 994.67(973.45,1015.88) |
| North Africa and Middle East | 235717.82(149529.35,352927.83) | 353585.22(219079.49,538849.65) | 13.10(5.14,19.30) | 3829.13(3798.31,3859.96) |
| South Asia | 750319.10(434940.16,1102366.71) | 954907.74(593786.46,1421944.65) | 9.94(1.35,15.94) | 6983.98(6735.32,7232.64) |
| Central Sub-Saharan Africa | 134895.66(43492.52,291095.55) | 182171.74(79115.02,418333.62) | 25.75(-22.36,118.83) | 1512.51(1475.20,1549.82) |
| Eastern Sub-Saharan Africa | 562502.40(142121.19,1176712.30) | 716734.93(242400.60,1485798.13) | 13.35(-10.42,45.31) | 4899.99(4739.30,5060.68) |
| Southern Sub-Saharan Africa | 63803.51(41792.79,97109.84) | 77690.63(50982.47,110902.64) | 9.89(-7.19,22.93) | 458.99(440.00,477.97) |
| Western Sub-Saharan Africa | 451623.42(120903.29,1131878.73) | 754881.66(279502.08,1730430.06) | 23.07(3.84,50.91) | 9844.21(9586.36,10102.06) |
| High-middle SDI | 941477.29(625654.11,1309786.48) | 693662.38(432190.86,1035712.60) | -15.61(-26.02,-3.79) | -7888.86(-8065.58,-7712.14) |
| High SDI | 744719.67(468695.86,1099158.15) | 770321.97(472107.05,1140343.11) | 2.64(0.09,4.49) | 695.53(630.71,760.36) |
| Low-middle SDI | 1015768.59(591906.62,1491181.20) | 1259326.37(775008.46,1846684.03) | 9.38(-1.29,17.16) | 7883.63(7690.06,8077.19) |
| Low SDI | 1065585.98(309522.07,2255995.70) | 1541577.30(573116.00,3044770.05) | 18.05(-0.09,48.17) | 15339.47(14830.22,15848.73) |
| Middle SDI | 1832647.00(1231731.77,2495439.57) | 1409841.90(878877.08,2106997.92) | -16.34(-27.56,-1.05) | -13537.93(-13955.26,-13120.60) |

Notes: AAPC, average annual percent change; CI, Confidence interval; DALY, disability-adjusted life years; GBD, Global Burden of Disease; SDI, Sociodemographic Index; UI, Uncertainty interval; URI, upper respiratory infections.

**Table S5**

The disease burden (ASRI, ASPR, ASMR, age-standardized DALY) of URIs across 204 countries and territories, and the changing trend of disease burden were analyzed across 204 countries and territories (top five and low five) from 1990 to 2021 year.

| Feature | Rank | Year | Index | Rate/ Number | Nations | Number (95%UI)/AAPC(95%CI) |
| --- | --- | --- | --- | --- | --- | --- |
| High | 1 | 2021 | incidence | rate | Greenland | 316881.49(280832.72, 355400.55) |
| High | 2 | 2021 | incidence | rate | Canada | 316139.22(281393.17, 354131.93) |
| High | 3 | 2021 | incidence | rate | United States of America | 295981.13(263871.79, 331046.61) |
| High | 4 | 2021 | incidence | rate | Kingdom of Thailand | 270056.59(236681.71, 303805.98) |
| High | 5 | 2021 | incidence | rate | Japan | 253469.12(224738.79, 286283.88) |
| Low | 1 | 2021 | incidence | rate | Republic of Tajikistan | 79718.98(70006.78, 90571.84) |
| Low | 2 | 2021 | incidence | rate | Kyrgyz Republic | 88838.66(78128.35, 102118.1) |
| Low | 3 | 2021 | incidence | rate | Republic of Uzbekistan | 93294.17(81819.94, 106531.35) |
| Low | 4 | 2021 | incidence | rate | Republic of Azerbaijan | 94979.33(83306.27, 109290.01) |
| Low | 5 | 2021 | incidence | rate | Islamic Republic of Mauritania | 96038.49(85184.31, 109617.66) |
| High | 1 | 2021 | incidence | Number | People's Republic of China | 1755242453(1548648295, 1977597229) |
| High | 2 | 2021 | incidence | Number | Republic of India | 1739214969(1525178087, 1999237408) |
| High | 3 | 2021 | incidence | Number | United States of America | 891500437(799101312, 984587944) |
| High | 4 | 2021 | incidence | Number | Republic of Indonesia | 580085491(511810095, 663233956) |
| High | 5 | 2021 | incidence | Number | Federative Republic of Brazil | 502050824(444098505, 570310120) |
| Low | 1 | 2021 | incidence | Number | Tokelau | 2858(2524, 3252) |
| Low | 2 | 2021 | incidence | Number | Republic of Niue | 3360(2968, 3814) |
| Low | 3 | 2021 | incidence | Number | Republic of Nauru | 25364(22068, 29356) |
| Low | 4 | 2021 | incidence | Number | Tuvalu | 26872(23661, 30508) |
| Low | 5 | 2021 | incidence | Number | Republic of Palau | 34712(30592, 39042) |
| High | 1 | 2021 | prevalence | rate | Greenland | 4386.75(3888.9, 4905.29) |
| High | 2 | 2021 | prevalence | rate | Canada | 4376.81(3901.14, 4902.77) |
| High | 3 | 2021 | prevalence | rate | United States of America | 4101.96(3661.93, 4571.78) |
| High | 4 | 2021 | prevalence | rate | Thailand | 3733.2(3270.01, 4192.52) |
| High | 5 | 2021 | prevalence | rate | Japan | 3513.53(3107.99, 3971.99) |
| Low | 1 | 2021 | prevalence | rate | Tajikistan | 1097.34(964.71, 1246.8) |
| Low | 2 | 2021 | prevalence | rate | Kyrgyzstan | 1223.2(1077.82, 1406.91) |
| Low | 3 | 2021 | prevalence | rate | Uzbekistan | 1284.45(1129.32, 1466.06) |
| Low | 4 | 2021 | prevalence | rate | Azerbaijan | 1307.62(1145.92, 1501.82) |
| Low | 5 | 2021 | prevalence | rate | Mauritania | 1322.9(1170.4, 1504.42) |
| High | 1 | 2021 | prevalence | Number | China | 24242865(21435341, 27352241) |
| High | 2 | 2021 | prevalence | Number | India | 23918746(20993672, 27441954) |
| High | 3 | 2021 | prevalence | Number | United States of America | 12321325(11062552, 13599013) |
| High | 4 | 2021 | prevalence | Number | Indonesia | 8034932(7082727, 9189900) |
| High | 5 | 2021 | prevalence | Number | Brazil | 6937662(6140400, 7877001) |
| Low | 1 | 2021 | prevalence | Number | Tokelau | 40(35, 45) |
| Low | 2 | 2021 | prevalence | Number | Niue | 46(41, 53) |
| Low | 3 | 2021 | prevalence | Number | Nauru | 351(306, 403) |
| Low | 4 | 2021 | prevalence | Number | Tuvalu | 372(327, 422) |
| Low | 5 | 2021 | prevalence | Number | Palau | 479.4(421.56, 539.63) |
| High | 1 | 2021 | Death | rate | Oman | 4.63(2.61, 9.49) |
| High | 2 | 2021 | Death | rate | Somalia | 3.02(0.03, 13.06) |
| High | 3 | 2021 | Death | rate | Central African Republic | 2.47(0.11, 8.07) |
| High | 4 | 2021 | Death | rate | Mozambique | 2.24(0.11, 6.14) |
| High | 5 | 2021 | Death | rate | South Sudan | 2.06(0.12, 7.05) |
| Low | 1 | 2021 | Death | rate | Bermuda | 0(0, 0) |
| Low | 2 | 2021 | Death | rate | Fiji | 0(0, 0) |
| Low | 3 | 2021 | Death | rate | United States Virgin Islands | 0(0, 0) |
| Low | 4 | 2021 | Death | rate | Greenland | 0(0, 0) |
| Low | 5 | 2021 | Death | rate | Northern Mariana Islands | 0(0, 0) |
| High | 1 | 2021 | Death | Number | Nigeria | 2469(317, 7001) |
| High | 2 | 2021 | Death | Number | China | 2193(1286, 5357) |
| High | 3 | 2021 | Death | Number | Ethiopia | 1440(77, 5185) |
| High | 4 | 2021 | Death | Number | India | 1094(204, 2460) |
| High | 5 | 2021 | Death | Number | Democratic Republic of the Congo | 1002(55, 3978) |
| Low | 1 | 2021 | Death | Number | Bermuda | 0(0, 0) |
| Low | 2 | 2021 | Death | Number | Tokelau | 0(0, 0) |
| Low | 3 | 2021 | Death | Number | Niue | 0(0, 0) |
| Low | 4 | 2021 | Death | Number | Greenland | 0(0, 0) |
| Low | 5 | 2021 | Death | Number | Cook Islands | 0(0, 0) |
| High | 1 | 2021 | DALY | rate | Central African Republic | 210(69, 500) |
| High | 2 | 2021 | DALY | rate | Federal Republic of Somalia | 208.21(43.82, 699.15) |
| High | 3 | 2021 | DALY | rate | Sultanate of Oman | 186.59(121.43, 338.76) |
| High | 4 | 2021 | DALY | rate | Republic of South Sudan | 175.11(53.27, 502.06) |
| High | 5 | 2021 | DALY | rate | Republic of Mozambique | 166.56(43.3, 379.12) |
| Low | 1 | 2021 | DALY | rate | Islamic Republic of Afghanistan | 36.96(22.47, 55.34) |
| Low | 2 | 2021 | DALY | rate | Georgia | 41.15(27.15, 60.03) |
| Low | 3 | 2021 | DALY | rate | Hashemite Kingdom of Jordan | 42.44(26, 63.56) |
| Low | 4 | 2021 | DALY | rate | Republic of Azerbaijan | 44.89(28.07, 71.2) |
| Low | 5 | 2021 | DALY | rate | Taiwan (Province of China) | 45.6(28.13, 68.44) |
| High | 1 | 2021 | DALY | Number | Republic of India | 650300(407403, 965503) |
| High | 2 | 2021 | DALY | Number | People's Republic of China | 649505(401570, 970569) |
| High | 3 | 2021 | DALY | Number | Federal Republic of Nigeria | 323479(131312, 678489) |
| High | 4 | 2021 | DALY | Number | United States of America | 306718(185873, 452270) |
| High | 5 | 2021 | DALY | Number | Republic of Indonesia | 204124(122775, 312731) |
| Low | 1 | 2021 | DALY | Number | Tokelau | 1(1, 2) |
| Low | 2 | 2021 | DALY | Number | Republic of Niue | 1(1, 2) |
| Low | 3 | 2021 | DALY | Number | Republic of Nauru | 9(5, 14) |
| Low | 4 | 2021 | DALY | Number | Tuvalu | 9(6, 14) |
| Low | 5 | 2021 | DALY | Number | Republic of Palau | 12(7, 18) |
| ascending | 1 | 1990-2021 | Incidence | rate | Rwanda | 476.022(442.640,509.405) |
| ascending | 2 | 1990-2021 | Incidence | rate | Guatemala | 268.579(259.101,278.058) |
| ascending | 3 | 1990-2021 | Incidence | rate | Maldives | 212.728(195.433,230.023) |
| ascending | 4 | 1990-2021 | Incidence | rate | Dominican Republic | 205.349(174.515,236.182) |
| ascending | 5 | 1990-2021 | Incidence | rate | Burundi | 196.930(166.214,227.646) |
| descending | 1 | 1990-2021 | Incidence | rate | United States of America | -1271.207(-1330.221,-1212.193) |
| descending | 2 | 1990-2021 | Incidence | rate | India | -401.183(-437.883,-364.484) |
| descending | 3 | 1990-2021 | Incidence | rate | South Africa | -348.102(-358.994,-337.211) |
| descending | 4 | 1990-2021 | Incidence | rate | Philippines | -338.739(-356.239,-321.239) |
| descending | 5 | 1990-2021 | Incidence | rate | Yemen | -318.947(-362.924,-274.969) |
| ascending | 1 | 1990-2021 | Prevalence | rate | Rwanda | 6.137(5.711,6.563) |
| ascending | 2 | 1990-2021 | Prevalence | rate | Guatemala | 3.996(3.837,4.155) |
| ascending | 3 | 1990-2021 | Prevalence | rate | Maldives | 2.990(2.711,3.269) |
| ascending | 4 | 1990-2021 | Prevalence | rate | Dominican Republic | 2.886(2.447,3.326) |
| ascending | 5 | 1990-2021 | Prevalence | rate | Burundi | 2.842(2.404,3.281) |
| descending | 1 | 1990-2021 | Prevalence | rate | United States of America | -17.074(-17.954,-16.194) |
| descending | 2 | 1990-2021 | Prevalence | rate | India | -5.130(-5.621,-4.638) |
| descending | 3 | 1990-2021 | Prevalence | rate | Philippines | -5.114(-5.376,-4.851) |
| descending | 4 | 1990-2021 | Prevalence | rate | South Africa | -4.613(-4.763,-4.463) |
| descending | 5 | 1990-2021 | Prevalence | rate | Yemen | -4.503(-5.138,-3.867) |
| ascending | 1 | 1990-2021 | Death | rate | Afghanistan | 0.003(0.003,0.003) |
| ascending | 2 | 1990-2021 | Death | rate | Qatar | 0.001(0.001,0.001) |
| ascending | 3 | 1990-2021 | Death | rate | Yemen | 0.001(0.001,0.001) |
| ascending | 4 | 1990-2021 | Death | rate | Grenada | 0.001(0.000,0.001) |
| ascending | 5 | 1990-2021 | Death | rate | Guyana | 0.001(0.000,0.001) |
| descending | 1 | 1990-2021 | Death | rate | Oman | -0.249(-0.260,-0.239) |
| descending | 2 | 1990-2021 | Death | rate | China | -0.096(-0.098,-0.095) |
| descending | 3 | 1990-2021 | Death | rate | Ethiopia | -0.057(-0.058,-0.056) |
| descending | 4 | 1990-2021 | Death | rate | Equatorial Guinea | -0.053(-0.054,-0.052) |
| descending | 5 | 1990-2021 | Death | rate | Taiwan (Province of China) | -0.039(-0.040,-0.038) |
| ascending | 1 | 1990-2021 | DALY | rate | Republic of Zimbabwe | 0.072(0.035,0.108) |
| ascending | 2 | 1990-2021 | DALY | rate | Islamic Republic of Afghanistan | 0.057(0.054,0.060) |
| ascending | 3 | 1990-2021 | DALY | rate | Republic of Maldives | 0.041(0.036,0.046) |
| ascending | 4 | 1990-2021 | DALY | rate | Grenada | 0.032(0.001,0.062) |
| ascending | 5 | 1990-2021 | DALY | rate | Independent State of Papua New Guinea | 0.031(0.028,0.033) |
| descending | 1 | 1990-2021 | DALY | rate | Sultanate of Oman | -9.552(-9.780,-9.323) |
| descending | 2 | 1990-2021 | DALY | rate | Federal Democratic Republic of Ethiopia | -3.407(-3.482,-3.332) |
| descending | 3 | 1990-2021 | DALY | rate | Republic of Liberia | -2.931(-3.243,-2.620) |
| descending | 4 | 1990-2021 | DALY | rate | People's Republic of China | -2.927(-2.967,-2.886) |
| descending | 5 | 1990-2021 | DALY | rate | Republic of Equatorial Guinea | -2.818(-2.871,-2.766) |

Notes: AAPC: average annual percent change; ASIR, age-standardized incidence rate; ASMR, age-standardized mortality rate; ASPR, age-standardized prevalence rate; CI, Confidence interval; DALYs, disability-adjusted life years; UI, Uncertainty interval; URI, upper respiratory infections.


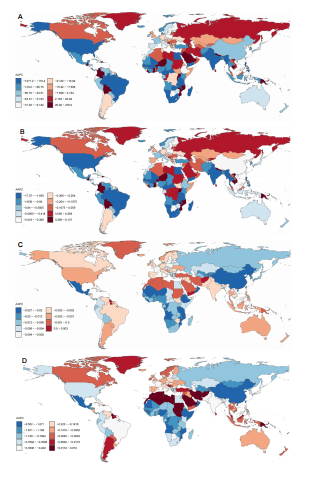


Figure S1. The changing trend (AAPC ) of ASR were analyzed across 204 countries and territories ( A: ASIR, B: ASPR. C: ASMR. D: Age-standardized DALY rate. Abbreviations: AAPC, average annual percent change; ASR, age-standardized rate; ASIR, age-standardized incidence rate; ASMR, age-standardized mortality rate; ASPR, age-standardized prevalence rate; DALYs, disability-adjusted life years; UI, Uncertainty interval; URI, upper respiratory infections).


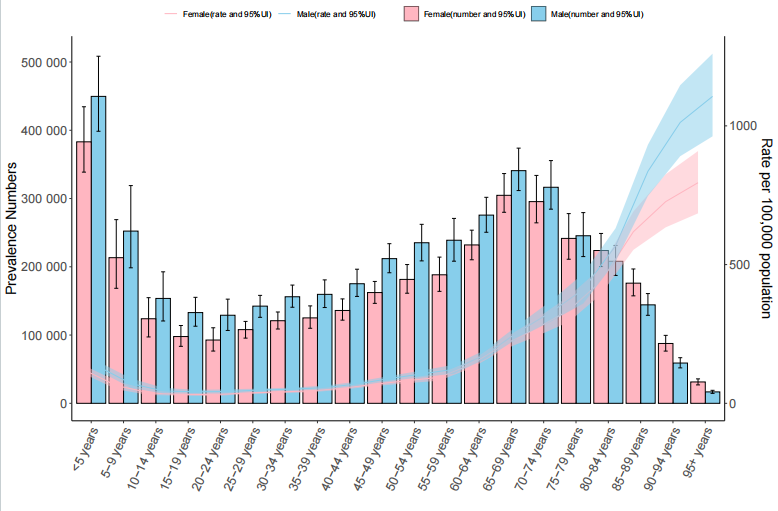


A


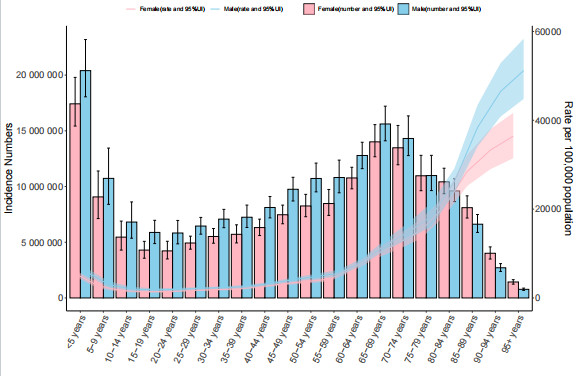

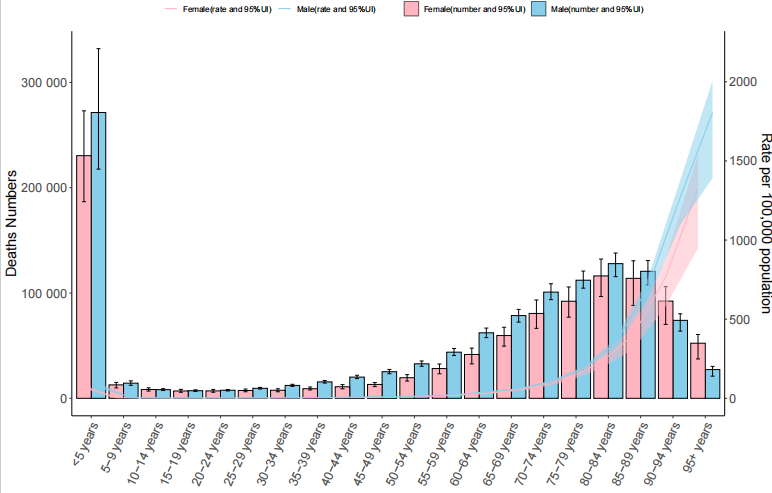


C

B


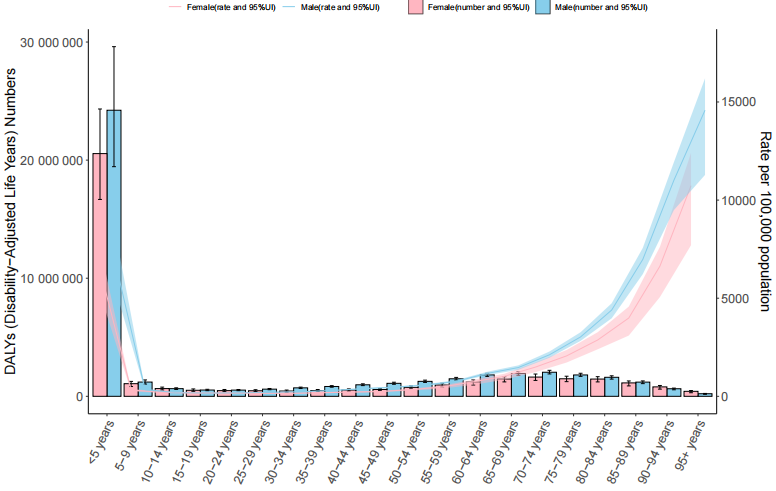


D

Figure S2. The ASRs of upper respiratory infections showed notable differences across age and gender distributions in 2021 year ( A: ASIR, B: ASPR. C: ASMR. D: Age-standardized DALY rate. Abbreviations: ASIR, age-standardized incidence rate; ASMR, age-standardized mortality rate; ASPR, age-standardized prevalence rate; DALYs, disability-adjusted life years; UI, Uncertainty interval).


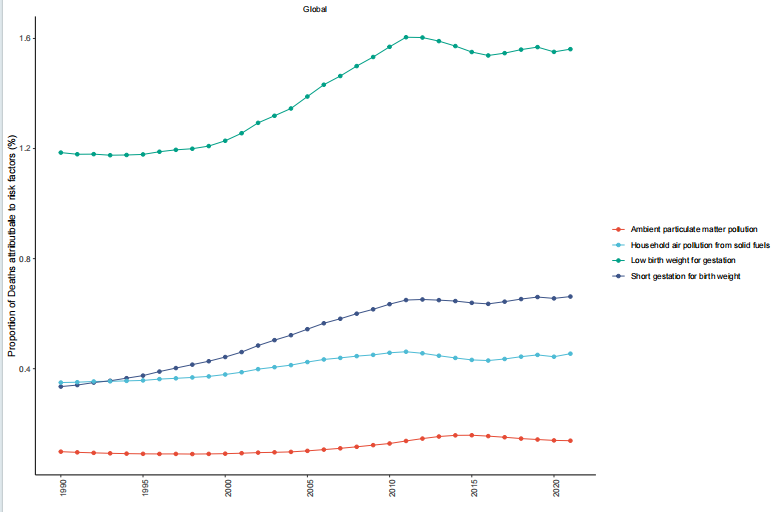


A


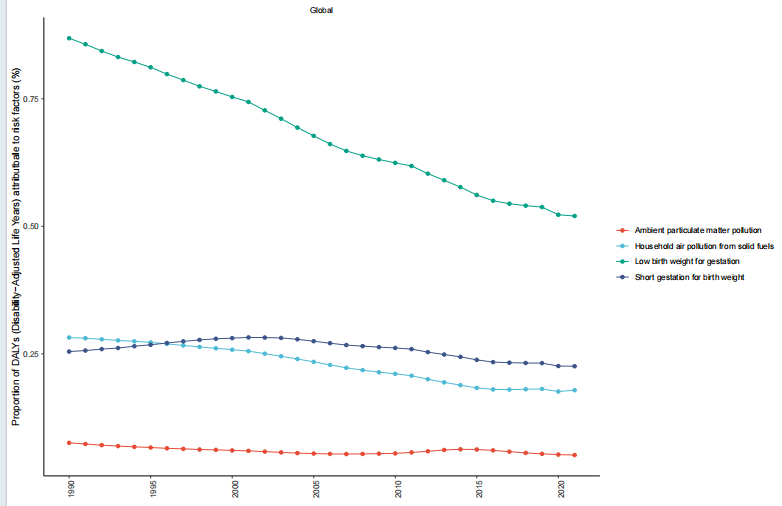


B

Figure S3: Trends in ASMR and age-standardized DALY rate attributed to upper respiratory infections from 1990 to 2021(A: ASMR. B:Age-standardized DALY rate. Abbreviations: ASIR, age-standardized incidence rate; ASMR, age-standardized mortality rate; DALYs, disability-adjusted life years; UI, Uncertainty interval).


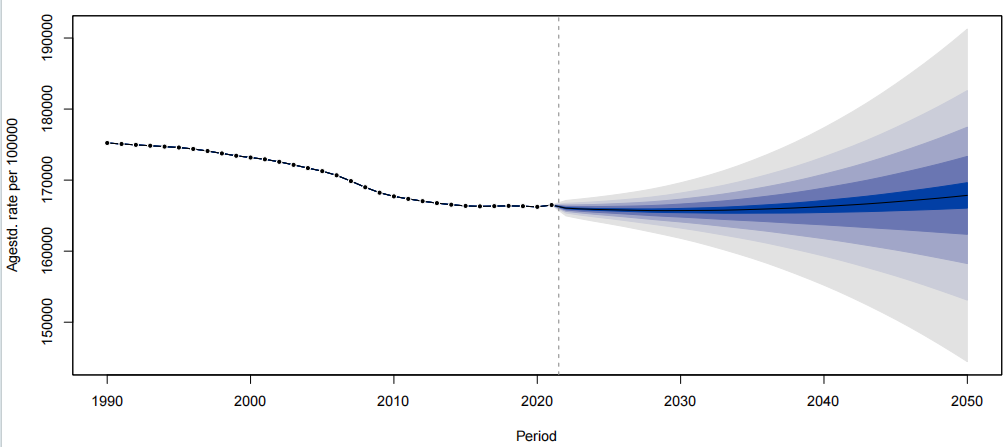


A


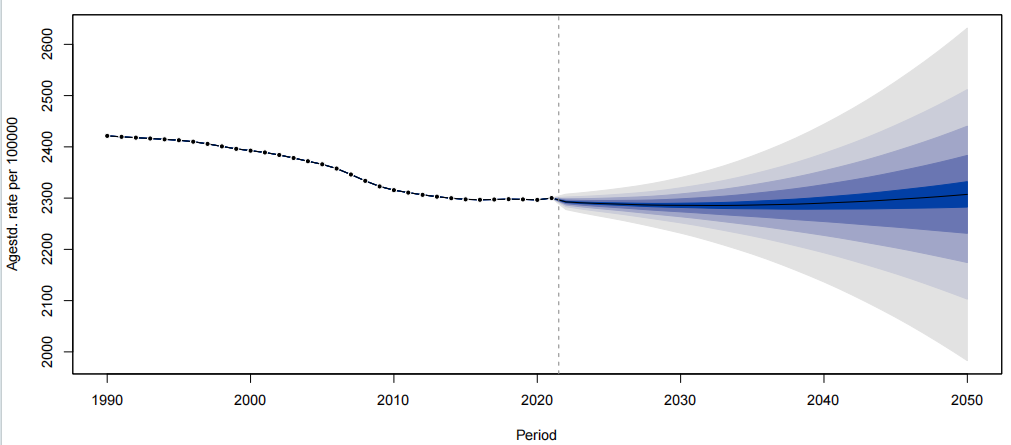


B


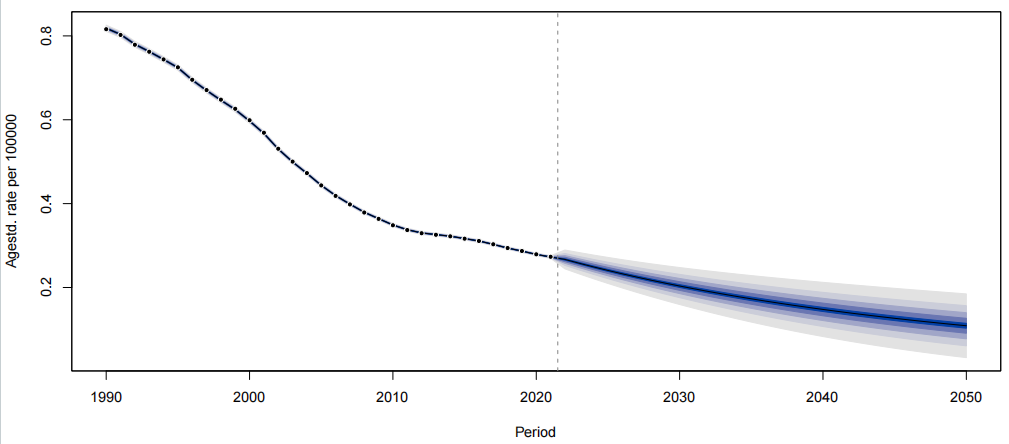


C


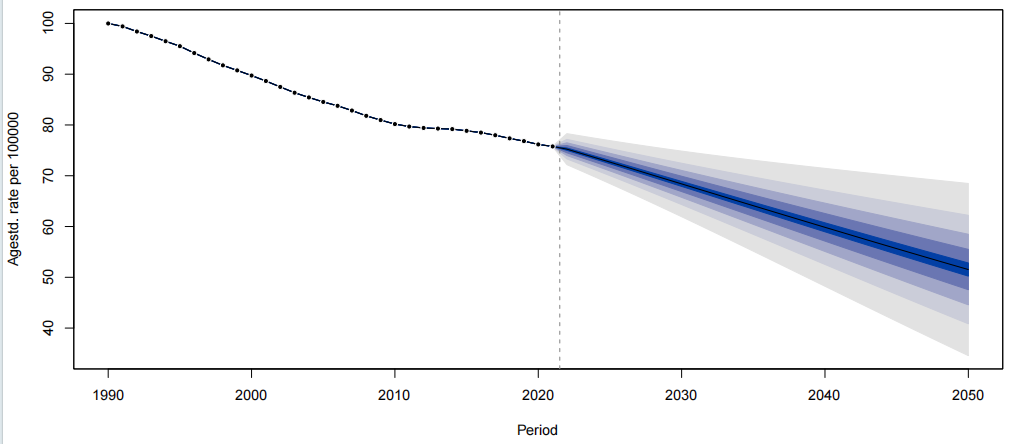


D

Figure S4: Prediction of global disease burden of upper respiratory infections from 2022–2050 years based on the BAPC model (A: ASIR, B: ASPR. C: ASMR. D: Age-standardized DALY rate. Abbreviations: ASIR, age-standardized incidence rate; ASMR, age-standardized mortality rate; ASPR, age-standardized prevalence rate; DALYs, disability-adjusted life years; UI, Uncertainty interval).
